# Structural and functional characterization of the gut microbiota in women with early pregnancy loss in Northwest China

**DOI:** 10.3389/fmicb.2026.1895733

**Published:** 2026-10-02

**Authors:** Hui Yao, Juanjuan Chen, Cai Liu, Juanjuan Huang, Ling Liu, Qi Wang, Fang Wang

**Affiliations:** 1 The Second School of Clinical Medicine, Lanzhou University, Lanzhou, Gansu, China; 2 Reproductive Medicine Center, Lanzhou University Second Hospital, Lanzhou, Gansu, China; 3 Cuiying Biomedical Research Center, Lanzhou University Second Hospital, Lanzhou, Gansu, China

**Keywords:** alpha diversity, beta diversity, gut microbiota, metagenomic sequencing, pregnancy loss

## Abstract

**Background:**

Early pregnancy loss (EPL) is a common adverse pregnancy outcome with a complex and incompletely understood etiology. Moreover, metagenomic studies of the gut microbiota in women with EPL in Northwest China remain limited.

**Objective:**

This study aimed to characterize differences in gut microbial composition and predicted functional potential between women with EPL (EPL group) and normal controls (NCs) using shotgun metagenomic sequencing.

**Methods:**

We collected fecal samples from 216 women in Northwest China (EPL group, *n* = 72; NCs, *n* = 144). Shotgun metagenomic sequencing was performed to analyze gut microbiota composition and assess the functional potential of the microbiota.

**Results:**

Our study revealed significant differences in the composition of the gut microbiota between the EPL group and NCs, as assessed by alpha and beta diversity analyses (*p* < 0.001). *Bacteroides* was significantly more abundant in the EPL group, whereas *Coprococcus*, *Bifidobacterium*, and *Roseburia* were significantly more abundant in NCs. Compared to NCs, the EPL group had higher relative abundances of species such as *Bacteroides stercoris*, *Bacteroides plebeius*, and *Bacteroides uniformis*, but lower relative abundances of *Bifidobacterium adolescentis*, *Megamonas funiformis*, and *Fusicatenibacter saccharivorans*. Partial correlation analysis showed that Bacteroidetes was positively correlated with EPL at the phylum level. At the genus level, *Odoribacter*, *Parabacteroides*, and *Haemophilus* were positively correlated with EPL, whereas *Corynebacterium*, *Lactobacillus*, and *Roseburia* were negatively correlated with EPL. Metabolic pathway analysis revealed that pathways such as L-rhamnose degradation I, L-arginine biosynthesis III and 2-oxobutanoate degradation I exhibited significant positive correlations with EPL. In contrast, pathways including purine nucleobases degradation I (anaerobic) and chitin derivatives degradation showed significant negative correlations with EPL.

**Conclusion:**

The results of our study revealed significant differences in gut microbiota composition and metabolic pathways between the EPL group and NCs, indicating that gut microbiome alterations are associated with EPL.

## 1 Introduction

Early pregnancy loss (EPL) refers to spontaneous pregnancy loss during the first trimester and affects approximately 15–20% of clinically recognized pregnancies worldwide (Yland et al., 2024; Quenby et al., 2021). An estimated 23 million miscarriages occur worldwide each year, equivalent to approximately 44 pregnancy losses every minute (Quenby et al., 2021). The etiology of EPL is complex and involves genetic, endocrine, metabolic, and immune factors, including thyroid disease, diabetes mellitus, and autoimmune conditions such as systemic lupus erythematosus and antiphospholipid syndrome (Sankoda et al., 2024; Chen X. et al., 2023; Keum et al., 2024; Sonehara et al., 2024). One such factor that has recently garnered attention is the role of the intestinal microbiome. Studies have confirmed that intestinal flora is involved in the regulation of the body’s immune, endocrine and metabolic functions (Dong and Mayer, 2024).

The gut microbiota is a complex ecosystem that contributes to host metabolism, immune regulation, and neuroendocrine signaling (Dong and Mayer, 2024; Koren et al., 2024). Dysbiosis of the gut microbiota has been linked to several gastrointestinal, cardiovascular, and respiratory diseases (Bijla et al., 2024; Wang et al., 2024; Grigorescu et al., 2023). The gut microbiota, by regulating the host’s metabolism and immunity, supports women’s health but may also be involved in reproductive disorders, including EPL (Marano et al., 2023; Xiao et al., 2024; Zhou Z. et al., 2024). Many scholars have noted that populations at high risk for EPL, such as endometriosis (EMs) or polycystic ovary syndrome (PCOS), differ significantly from healthy populations in terms of the type, distribution, and amount of gut microbiota (Zhou M. et al., 2024; Tang et al., 2024). Research has shown that the gut microbiota can influence the immune system and promote chronic inflammation, which is linked to adverse pregnancy outcomes, including preterm birth and preeclampsia (Lei et al., 2024; Xu et al., 2024). In fact, women with active IBD and autoimmune thyroiditis may have a higher risk of EPL (Grigorescu et al., 2023; Song et al., 2024), which also suggests a role for gut flora in inflammatory mechanisms. Previous studies suggest that ecological imbalances in the gut, vagina, or uterus may affect estrogen metabolism and immune homeostasis and may contribute to EPL (Li et al., 2024; Zhu et al., 2023; Giannella et al., 2023). In addition, the gut microbiota can influence the production and bioavailability of metabolites in the body, such as short-chain fatty acids (SCFAs) (Gao et al., 2024). Studies have shown that SCFAs are important for maintaining the integrity of the intestinal barrier and modulating the immune response, and that SCFA deficiency may impair placental function and lead to adverse pregnancy outcomes (Ruiz-Triviño et al., 2023). Currently, the relationship between gut flora and EPL remains largely unexplored. Moreover, existing studies have generally involved small sample sizes and limited sequencing resolution. Therefore, it is necessary to explore the differential changes in the distribution of gut flora in the EPL group.

In this study, we collected fecal samples from 144 NCs and 72 women with EPL from the high-altitude city of Lanzhou for analysis, representing the first metagenomic study of EPL conducted in Northwest China. This study provides insight into the association between the gut microbiota and EPL and a foundation for identifying microbial markers associated with EPL.

## 2 Materials and methods

### 2.1 Participants

A total of 216 participants were recruited for the study between January 2022 and December 2023, comprising 72 women with EPL and 144 NCs. The demographic and clinical characteristics of the enrolled participants are detailed in Supplementary Table S1. Sample size was estimated using a standard case–control formula, and a 1:2 case-to-control ratio was adopted to enhance statistical power given the clinical constraints in recruiting early pregnancy loss cases. Women with EPL were included if they met the following criteria: (Yland et al., 2024) aged 18–45 years; (Quenby et al., 2021) a history of at least one early clinical pregnancy loss occurring during the first trimester; (Sankoda et al., 2024) naturally conceived; and (Chen X. et al., 2023) with available information on age, BMI, race, education, number of previous pregnancy losses, and blood pressure. NCs were defined as women with at least one uncomplicated pregnancy and no history of miscarriage, preeclampsia, gestational diabetes mellitus, gestational hypertension, preterm delivery, intrauterine growth restriction, or other pregnancy-related complications. At enrollment, trained research staff used a structured questionnaire to obtain a detailed medication history. Individuals reporting antibiotic use within the preceding 2 months or probiotic use within the preceding 1 month were excluded (Martínez-Sánchez et al., 2022; Scanu et al., 2024). Compliance with these criteria was assessed from the structured medication history. This screening approach is consistent with standard practice in large-scale microbiome studies and helps reduce potential confounding from recent antibiotic- or probiotic-related disturbances to the gut microbiota. The characteristics of the EPL and NC groups are shown in Table 1. Fecal samples were collected from all participants and subjected to metagenomic sequencing.

**Table 1:** Baseline characteristics of the EPL group and NCs.

Characteristics	NCs (*n* = 144)	EPL (*n* = 72)	*p*
Age, *n* (%), years			<0.001
≧22& < 30	21 (14.6)	28 (38.9)	
≧30& < 35	60 (41.7)	31 (43.1)	
≧35 &≦42	63 (43.8)	13 (18.1)	
BMI, *n* (%), kg/m^2^			0.339
<18.5	12 (8.3)	3 (4.2)	
≧18.5& < 28	125 (86.8)	63 (87.5)	
≧28	7 (4.9)	6 (8.3)	
Race, *n* (%)			0.246
Han	139 (96.5)	68 (94.4)	
Hui	3 (2.1)	1 (1.4)	
Zang	0 (0.0)	2 (2.8)	
Others	2 (1.4)	1 (1.4)	
Education, *n* (%)			<0.001
University degree or less	15 (10.4)	23 (31.9)	
University degree or above	129 (89.6)	49 (68.1)	
No_PL, *n* (%)			<0.001
0	144 (100.0)	0 (0.0)	
1	0 (0.0)	15 (20.8)	
2	0 (0.0)	36 (50.0)	
3	0 (0.0)	15 (20.8)	
≧4	0 (0.0)	6 (8.3)	
S_pressure (median [IQR]),mmHg	110.00 [103.75, 115.00]	115.00 [106.75, 123.00]	0.003
D_pressure (median [IQR]),mmHg	72.00 [65.00, 76.00]	72.00 [67.00, 78.00]	0.329
WHR, median [IQR]	0.82 [0.78, 0.85]	0.87 [0.83, 0.92]	<0.001

### 2.2 Sample collection

All participants (EPL group and NCs) were concurrently recruited and sampled over the study period, and fecal samples were collected and processed using identical standardized procedures to minimize potential time-related biases in microbiota composition. For women in the EPL group, fecal samples were collected after the pregnancy-loss event at enrollment. Fecal samples were collected by having participants defecate onto a sterile pad. Using a sterile spoon, approximately 2 g of feces was placed into a sterile 2 mL tube pre-filled with a protective solution. Samples were transported under cryogenic conditions and stored at −80 °C until further processing.

### 2.3 Metagenomic sequencing and analysis

To investigate the microbial taxonomic profiles associated with EPL, we performed shotgun metagenomic sequencing on fecal samples collected from 72 women in the EPL group and 144 NCs. The stool specimens were submitted to MyGenostics Co. Ltd. (Beijing, China) for analysis. Microbial DNA was extracted and purified from each sample. A sequencing library was prepared using the NEXTflex Rapid DNA Sequencing Kit (Bioo Scientific, USA) following the manufacturer’s protocol. Library quality was assessed using Qubit 2.0, Agilent 2,100, and qPCR before sequencing on an Illumina NovaSeq 6,000 platform (paired-end, 2 × 150 bp). Raw reads were processed using MOCAT (version 2.0) to remove low-quality and short reads. Clean reads from each sample were assembled using MOCAT to obtain contigs >300 bp. The assembled sequences were aligned to the MicroNR database using Bowtie2 (version 2.5.4) for taxonomic annotation, and gene abundance tables were merged into a species-level abundance matrix. Linear discriminant analysis effect size (LEfSe) was applied to identify taxa with significantly different abundances between the EPL group and NCs, with a significance threshold of *p* < 0.05.

### 2.4 Diversity analysis

Alpha diversity (richness, Shannon index, Simpson’s index, and inverse Simpson’s index) was calculated using the diversity function from the R package vegan (version 4.0.3). Beta diversity was assessed based on Bray–Curtis distance using principal coordinate analysis (PCoA) implemented with the ape package (pcoa function, correction = “none”), and the distance matrix was also generated via vegan. Permutational multivariate analysis of variance (PERMANOVA) was performed using the adonis function (1,000 permutations) on genus- and species-level abundance profiles to evaluate the effect of group (EPL group vs. NCs) on the gut microbiome.

### 2.5 Partial correlation analysis

To account for potential confounding, partial spearman correlation analyses were performed using the R package ppcor. Associations between EPL status and the abundance profiles of microbial taxa (phylum, genus, and species) and functional pathways were evaluated while controlling for education level, age, and waist-to-hip ratio (WHR). For these association analyses, *p* values were adjusted for multiple testing using the Benjamini–Hochberg method, and adjusted *p* < 0.05 was considered statistically significant.

### 2.6 Functional profiling and pathway analysis

Functional profiles were generated from the metagenomic sequencing data using the HMP Unified Metabolic Analysis Network (HUMAnN, version 3.0) (Beghini et al., 2021). HUMAnN maps gene families to metabolic pathways in the MetaCyc database and produces pathway abundance tables. Per-sample pathway abundance tables were combined using HUMAnN_join_tables and converted to relative abundances using HUMAnN_renorm_table with the --units relab option. This total-sum scaling procedure divides each pathway abundance by the total community-level pathway abundance within the corresponding sample. The normalized abundance of each pathway was compared between the EPL and NC groups using one-way analysis of variance (ANOVA). The resulting *p* values were adjusted for multiple testing using the Benjamini–Hochberg method, and adjusted *p* < 0.05 was considered statistically significant. Gene Ontology (GO) profiles were generated by regrouping HUMAnN 3.0 gene-family abundances into molecular function (MF), biological process (BP), and cellular component (CC) categories.

### 2.7 Statistical analysis

Between-group differences in the relative abundances of microbial taxa at the phylum, genus, and species levels were assessed using the Wilcoxon rank-sum test, followed by Storey’s FDR correction. Functional pathway differences were analyzed separately using one-way ANOVA with Benjamini–Hochberg FDR correction, as described above. Partial Spearman correlations were used to evaluate associations of microbial taxa and functional pathways with EPL status after adjustment for education level, age, and WHR. Normally distributed continuous variables are presented as the mean ± standard deviation and were compared using Student’s t-test; non-normally distributed continuous variables were compared using the Mann–Whitney U test. All tests were two-sided, and *p* < 0.05 was considered statistically significant. Statistical analyses were performed using RStudio software (version 1.4.1717).

## 3 Results

### 3.1 Microbial diversity and differential taxonomic abundance between the EPL group and NCs

The distribution and diversity of the gut microbiota in each sample are summarized in Supplementary Table S2. To assess differences in microbial community composition between the EPL and NC groups, we performed beta diversity analysis based on Bray-Curtis distances. PCoA revealed significant separation between the two groups at both the genus level (R^2^ = 0.087, *p* < 0.001) and the species level (R^2^ = 0.047, *p* < 0.001) (Figure 1A; Supplementary Figure S1). Alpha diversity based on the Shannon, Simpson, and Inverse Simpson indices showed lower genus- and species-level diversity in the EPL group (Figure 1B; Supplementary Table S3, all *p* < 0.01).

**Figure 1:**
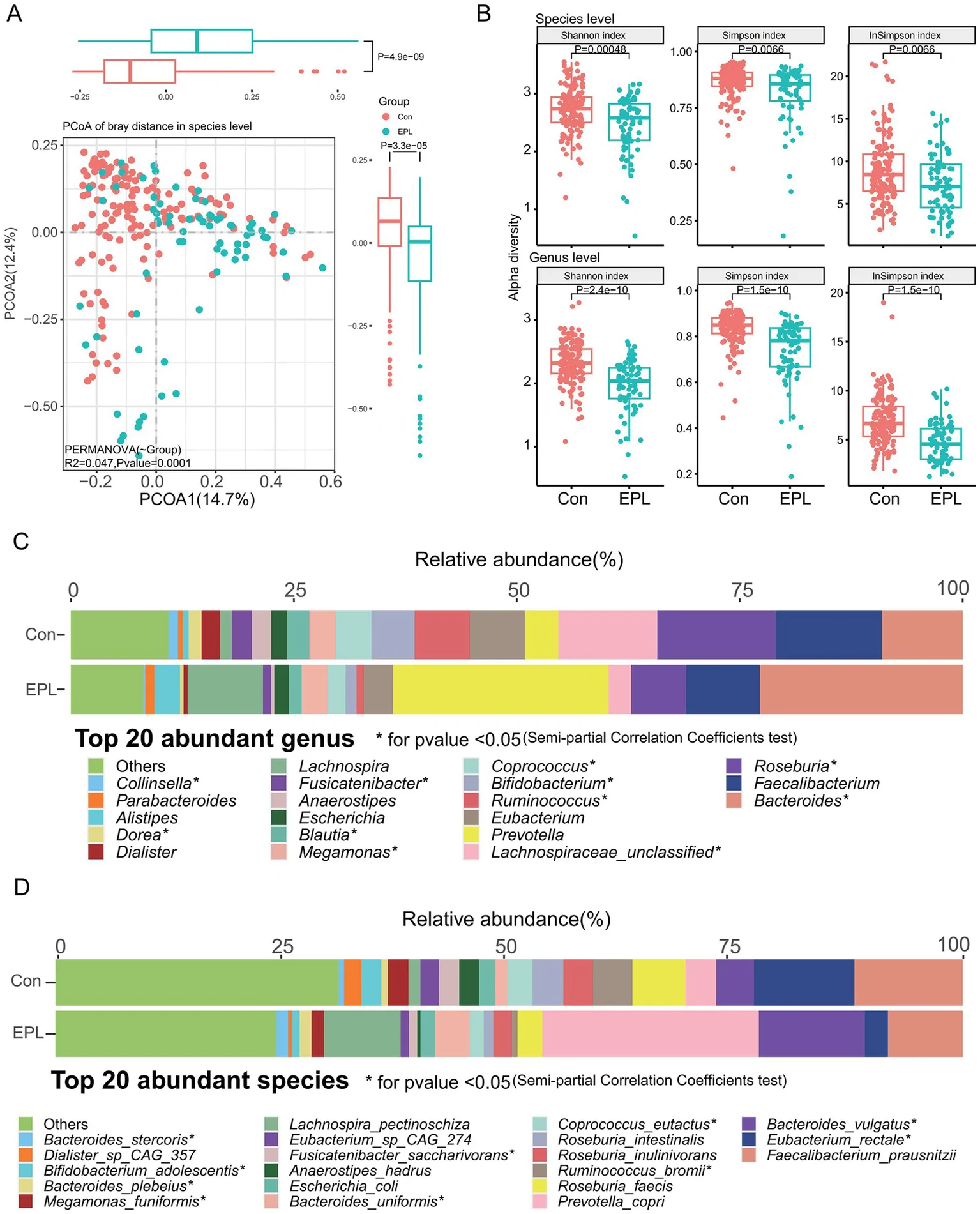
Gut microbial alterations associated with EPL. **(A)** PCoA showing significant differences in gut microbiota composition at the species level. **(B)** Genus-level alpha diversity assessed using the Shannon, Simpson, and inverse Simpson indices differed significantly between the EPL group and NCs. **(C,D)** The top 20 genera and species with significant differences between the EPL group and NCs. *p < 0.05 in panels **(C,D)**.

Subsequently, we analyzed the richness of the gut microbiota at the phylum, genus, and species levels (Supplementary Tables S4–S6), and compared the differences among the most abundant phyla, the top 20 most abundant genera, and the top 20 most abundant species.

The fecal microbiota of the EPL group was dominated by *Proteobacteria, Bacteroidetes,* and *Firmicutes* at the phylum level, whereas the fecal microbiota of NCs was dominated by *Actinobacteria, Bacteroidetes,* and *Firmicutes* (Supplementary Figure S1A). The relative abundances of *Firmicutes* and *Actinobacteria* were significantly lower in the EPL group than in the NC group, whereas the relative abundance of *Bacteroidetes* was significantly higher in the EPL group than in NCs (*p* < 0.001) (Supplementary Figure S1B). Among the 20 most abundant genera, *Coprococcus, Bifidobacterium, Roseburia*, unclassified Lachnospiraceae*, Collinsella, Dorea, Fusicatenibacter, Ruminococcus*, *Blautia*, and *Megamonas* were significantly more abundant in NCs, while *Bacteroides* was significantly more abundant in the EPL group. The high-abundance genera, including *Parabacteroides, Alistipes, Dialister, Lachnospira, Anaerostipes, Escherichia, Eubacterium*, *Prevotella*, and *Faecalibacterium*, showed no significant differences in their abundances between the two groups (Figure 1C).

Among the 20 most abundant species, the relative abundances of *Bifidobacterium adolescentis, Megamonas funiformis, Fusicatenibacter saccharivorans, Coprococcus eutactus, Ruminococcus bromii* and *Eubacterium rectale* were higher in NCs than in the EPL group, whereas *Bacteroides stercoris, Bacteroides plebeius, Bacteroides uniformis* and *Bacteroides vulgatus* were higher in the EPL group than in NCs. *Dialister* sp. CAG-357*, Lachnospira pectinoschiza, Eubacterium* sp. CAG-274*, Anaerostipes hadrus, Escherichia coli, Roseburia intestinalis, Roseburia inulinivorans, Roseburia faecis, Prevotella copri* and *Faecalibacterium prausnitzii* were present in both groups, with no significant differences in their abundances between the two groups (Figure 1D).

### 3.2 Partial correlation analysis of microbial taxa associated with EPL

To reduce potential confounding by education level, age, and WHR, we performed partial Spearman correlation analysis to examine the correlations between EPL and gut microbial taxa. At the genus level, *Bacteroides*, *Odoribacter*, *Parabacteroides* and *Haemophilus* showed positive correlations with EPL. Conversely, *Corynebacterium*, *Lactobacillus*, *Roseburia*, *Agathobaculum*, *Actinomyces*, *Mogibacterium*, *Ruminococcus*, *Fusicatenibacter*, *Coprococcus*, *Gemmiger*, *Blautia*, *Dorea*, *Megamonas*, *Bifidobacterium* and *Collinsella* showed negative correlations with EPL (Figure 2A). Among these, *Bacteroides* exhibited the strongest positive correlation with EPL, whereas *Collinsella* showed the strongest negative correlation.

**Figure 2:**
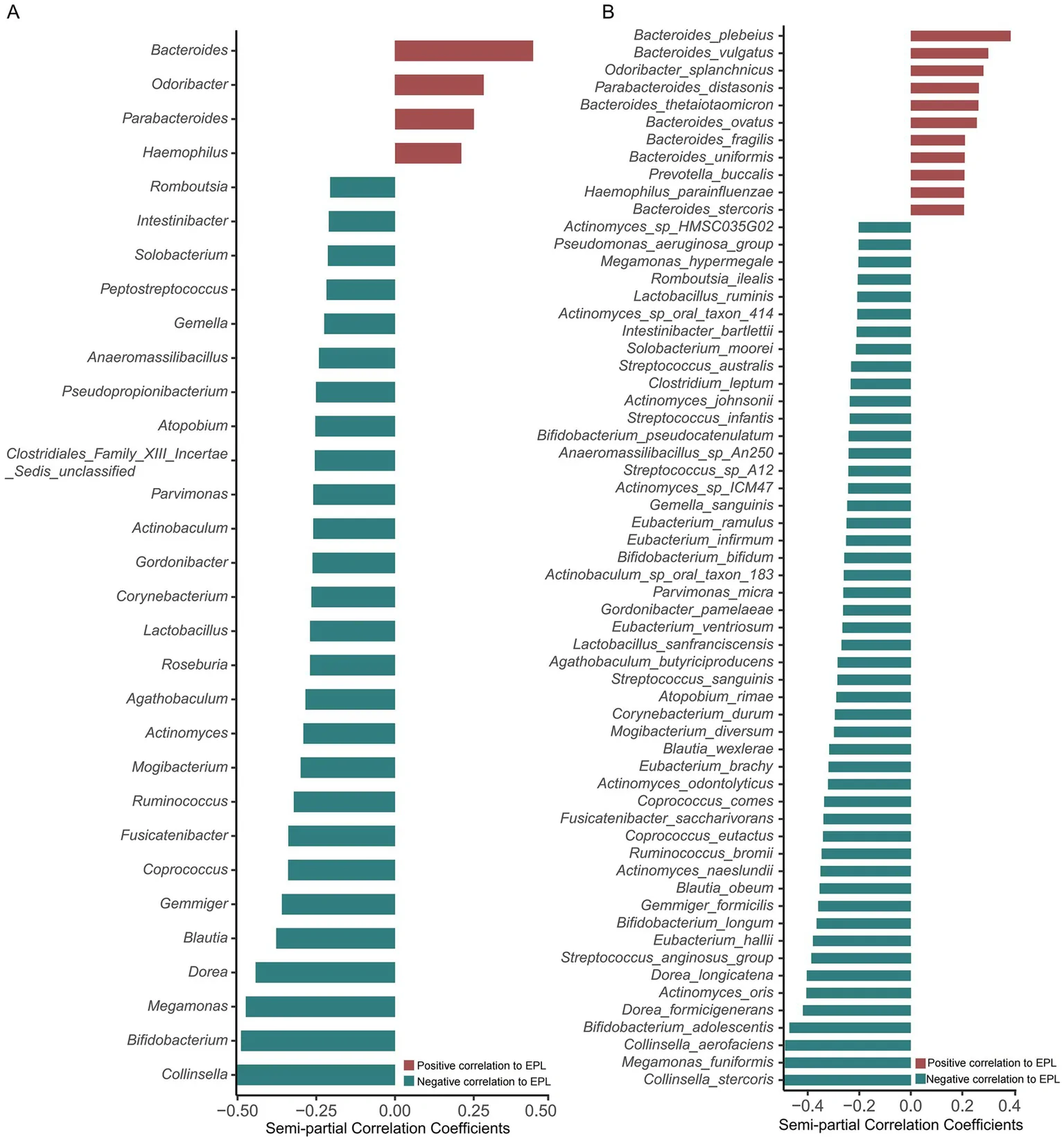
Gut microbial taxa significantly correlated with EPL. **(A,B)** Taxa significantly correlated with EPL and their relative abundances at the genus and species levels.

At the species level, several species—including *Bacteroides plebeius, Bacteroides vulgatus, Odoribacter splanchnicus, Parabacteroides distasonis, Bacteroides thetaiotaomicron, Bacteroides ovatus, Bacteroides fragilis, Bacteroides uniformis, Prevotella buccalis, and Haemophilus parainfluenzae*—showed positive correlations with EPL. In contrast, *Coprococcus eutactus, Ruminococcus bromii, Actinomyces naeslundii, Blautia obeum, Gemmiger formicilis, Bifidobacterium longum, Eubacterium hallii, Streptococcus anginosus* group*, Dorea longicatena, Actinomyces oris, Dorea formicigenerans, Bifidobacterium adolescentis, Collinsella aerofaciens, Megamonas funiformis,* and *Collinsella stercoris* were significantly negatively correlated with EPL (Figure 2B). Among these, *Bacteroides plebeius* showed the strongest positive correlation with EPL, while *Collinsella stercoris* showed the strongest negative correlation at the species level.

### 3.3 Identification of gut microbial functional capacity associated with EPL

HUMAnN 3.0 was used to analyze the metabolic pathways of the gut microbiota (Figure 3). PCoA analysis based on Bray–Curtis distances of pathway profiles revealed a significant difference in metabolic pathways between the two groups (Supplementary Figure S3, *p* = 0.016). A total of 40 pathways were significantly overrepresented in the microbiomes of the EPL group (Supplementary Figure S3). The 10 pathways most strongly positively correlated with EPL were: L-rhamnose degradation I, L-arginine biosynthesis III (via N-acetyl-L-citrulline), 2-oxobutanoate degradation I, thiamine diphosphate salvage II, L-histidine degradation I, L-histidine degradation III, pyridoxal 5′-phosphate biosynthesis I, lipid IVA biosynthesis (*P. putida*), lipid IVA biosynthesis (*E. coli*), and the superpathway of pyridoxal 5′-phosphate biosynthesis and salvage. In contrast, the prominent pathways negatively correlated with EPL included purine nucleobases degradation I (anaerobic), chitin derivatives degradation, stachyose degradation, L-arginine biosynthesis I (via L-ornithine), sucrose biosynthesis II, L-arginine biosynthesis II (acetyl cycle), starch biosynthesis, the *Bifidobacterium* shunt, and lactose and galactose degradation I.

**Figure 3:**
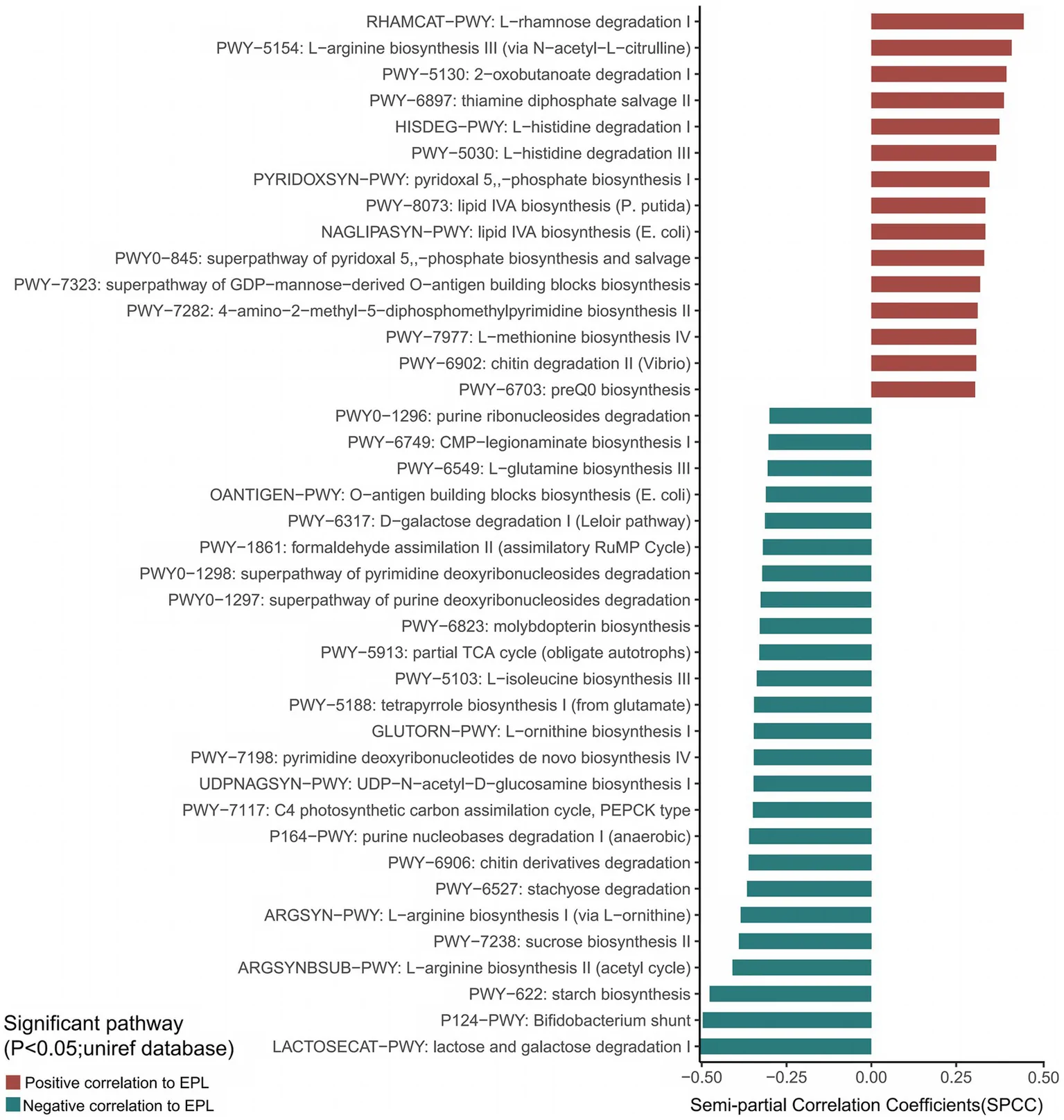
Predicted metagenomic functional pathways significantly correlated with EPL.

Additionally, we performed GO enrichment analysis to further evaluate the relationships between gut microbial species and functional annotations in the EPL group at the molecular function (MF), cellular component (CC), and biological process (BP) levels. PCoA analysis based on Bray–Curtis distances of GO term profiles revealed significant separation between the two groups (Supplementary Figure S4, *p* = 0.0018, PERMANOVA). The top 6 GO terms negatively associated with EPL at the MF level were: galactose-6-phosphate isomerase activity, glutaminyl-tRNA synthase (glutamine-hydrolyzing) activity, phosphoketolase activity, fructose-6-phosphate phosphoketolase activity, betaine reductase activity, and glycine reductase activity. At the CC level, the top 6 GO terms negatively associated with EPL were: ficolin-1-rich granule lumen, secretory granule lumen, glutamyl-tRNA (Gln) amidotransferase complex, proton-transporting V-type ATPase (V0 domain), tRNA (m1A) methyltransferase complex, and bacterial nucleoid. At the BP level, the top 6 GO terms negatively associated with EPL were: lactose catabolic process via tagatose-6-phosphate, glyceraldehyde-3-phosphate biosynthetic process, neutrophil degranulation, regulation of translational fidelity, N-terminal protein amino acid acetylation, and ribosome disassembly (Supplementary Figure S5).

## 4 Discussion

To our knowledge, this is the first study to investigate the correlation between the gut microbiota and early pregnancy loss (EPL) in Northwest China using metagenomic sequencing. In our study, PCoA analysis based on Bray–Curtis distances revealed a clear separation in gut microbiota composition between the EPL group and NCs, consistent with previous reports demonstrating significant microbial community differences between these groups (Gao et al., 2021; Liu et al., 2021). Alpha diversity indices, including the Shannon, Simpson, and inverse Simpson indices, showed that both species- and genus-level diversities were significantly lower in the EPL group than in NCs, which aligns with the findings of Liu et al. (2021).

At the phylum level, *Proteobacteria, Bacteroidetes,* and *Firmicutes* were the predominant phyla in the fecal microbiota of the EPL group, whereas *Actinobacteria, Bacteroidetes* and *Firmicutes* dominated in NCs. Compared with NCs, the EPL group exhibited lower abundances of *Firmicutes* and *Actinobacteria* but a higher abundance of *Bacteroidetes*, which aligns with previous reports of reduced *Actinobacteria* in the EPL group (Chen Y. et al., 2023). However, Chen Y. et al. (2023) reported a lower abundance of *Bacteroidetes* in the EPL group, while Gao et al. (2021) found higher *Firmicutes* abundance in the EPL group—findings that contradict our results. We speculate that these discrepancies may be attributable to differences in geographical region and dietary habits, as dietary patterns are known to profoundly shape gut microbial composition at the phylum level.

At the genus level, several taxa associated with SCFA production and intestinal homeostasis, including the genera *Coprococcus*, *Fusicatenibacter*, *Ruminococcus*, *Blautia*, and *Dorea*, as well as members of the family Lachnospiraceae, were found to be less abundant in the EPL group (Nonejuie et al., 2024; Gryaznova et al., 2024; Kemp et al., 2024; Yang et al., 2023). The reduced abundance of these taxa may indicate a less favorable gut environment, potentially contributing to disease pathogenesis. *Bifidobacterium,* a genus commonly found in the human gastrointestinal tract, is known for its probiotic properties and its ability to ferment carbohydrates into lactic acid (Hindle et al., 2025). Both animal and clinical studies have demonstrated that *Bifidobacterium* supplementation restores gut barrier function, reduces inflammation, and improves pregnancy outcomes, supporting its protective role against recurrent early pregnancy loss (Tersigni et al., 2024; Faas et al., 2025). Other genera enriched in the general population may be involved in regulating host metabolism.

Kemp et al. (2024) also identified *Roseburia* as a dominant genus in healthy individuals, a finding consistent with our results. This observation is also consistent with previous studies (Tanca et al., 2024; Liu et al., 2023; Mohebali et al., 2023). Experimental evidence further suggests that *Roseburia intestinalis* may relieve gestational intrahepatic cholestasis in rats via the bile acid/FXR–FGF15 axis (Sun et al., 2023). Changes in *Collinsella* abundance have been associated with insulin-related metabolic changes (Zhang et al., 2023); however, the metabolic significance of its higher abundance in NCs remains uncertain. Notably, the relative abundance of *Bacteroidetes* was significantly higher in the EPL group. Dietary patterns are known to influence members of the phylum *Bacteroidetes*, although the direction of these associations varies according to dietary composition, host population, and taxonomic level (Di Rosa et al., 2023; Schoonakker et al., 2025). Therefore, the enrichment of *Bacteroidetes* observed in the EPL group might partly reflect differences in dietary habits between groups; however, because dietary intake was not assessed, this interpretation remains speculative. Thus, the depletion of these SCFA-associated genera in the EPL group may be associated with reduced microbial SCFA-producing potential, impaired immune regulation, and a pro-inflammatory state relevant to early pregnancy loss. These associations may extend beyond the intestinal lumen and involve the proposed gut-uterus axis; however, their temporal and causal relationships remain to be established.

At the species level, *Fusicatenibacter saccharivorans* has been associated with bile acid metabolism and related enzyme activities (Jia et al., 2024). *Ruminococcus bromii* may support butyrate production indirectly through resistant-starch degradation and microbial cross-feeding, whereas *Eubacterium rectale* is an important butyrate-producing bacterium. Butyrate can reduce inflammation, support gut barrier integrity, and promote immune tolerance (Pickens and Cockburn, 2024; Karim et al., 2024)—benefits that may be lost in the EPL group. A relative increase in *Bacteroides stercoris*, *Bacteroides plebeius*, *Bacteroides uniformis* and *Bacteroides vulgatus* was observed in the EPL group.

In the EPL group, L-rhamnose degradation and L-arginine biosynthesis III were positively associated with EPL, whereas sucrose and starch biosynthesis were negatively associated. These associations suggest altered microbial carbohydrate and amino-acid metabolic potential, but their physiological consequences require experimental validation. At the predicted molecular-function level, negative associations with EPL were observed for galactose-6-phosphate isomerase (indicating disrupted galactose metabolism), glutaminyl-tRNA synthase (protein synthesis), phosphoketolase and fructose-6-phosphate phosphoketolase (energy/nucleotide synthesis), as well as betaine and glycine reductases (one-carbon/redox metabolism). At the cellular-component level, secretory-granule and ficolin-1-rich-granule lumens were negatively associated with EPL. Although complement is a major effector of innate immunity (Mastellos et al., 2024), these microbial functional annotations do not establish altered host complement or neutrophil activity, and their biological significance requires further investigation. At the biological process level, the lactose catabolic process via tagatose-6-phosphate was also negatively associated, further indicating altered predicted carbohydrate metabolism. These findings suggest an association between microbial dysbiosis and altered metabolic potential in EPL.

This study provides new insights into the association between the gut microbiome and early pregnancy loss. Its strengths are primarily reflected in the following aspects: First, this study systematically characterized the structural changes in the gut microbiome and abnormalities in metabolic pathways among individuals with early pregnancy loss in Northwest China. Second, the differential functional features identified (such as galactose-6-phosphate isomerase activity and glutaminyl-tRNA synthase activity), together with the microbial community characteristics, provide a basis for future studies of microbial features associated with EPL. However, several limitations should be acknowledged. First, genetic and environmental factors that may influence EPL were not comprehensively assessed. Age, education level, and WHR differed between the EPL and NC groups. Although these variables were controlled in the partial correlation analyses, covariate adjustment cannot fully eliminate residual confounding or limitations arising from incomplete covariate overlap. Future studies should use prospective recruitment with closer matching or larger samples. Second, the cross-sectional design precludes causal inference, and the observed microbial alterations may be either contributors to or consequences of EPL. Finally, because fecal samples from the EPL group were collected after the pregnancy-loss event and no paired pre-event samples were available, residual post-loss confounding due to inflammatory responses, psychological stress, dietary changes, or other unmeasured factors cannot be excluded. Prospective longitudinal studies with sampling before and after pregnancy outcomes are needed to establish temporal direction.

## 5 Conclusion

In summary, this study identifies an association between gut microbiota dysbiosis and EPL and provides a basis for future prospective and mechanistic studies.

## Data Availability

The metagenomic sequencing data that support the findings of this study are openly available in the NCBI BioProject database (https://www.ncbi.nlm.nih.gov/) under accession number PRJNA1390327.
